# Draft whole-genome sequence of *Aureobasidium pullulans* Y2311-1, a producer of poly(β-L-malic acid)

**DOI:** 10.1128/mra.00850-25

**Published:** 2026-08-17

**Authors:** Hao Tang, Ting Xue, Teng Xu, Jianghao Chen, Meimei Fu, Yiwen Jiang, Wenwen Tang, Jinshan Guo

**Affiliations:** 1 CAS Key Laboratory of High-Performance Synthetic Rubber and Its Composite Materials, Changchun Institute of Applied Chemistry, Chinese Academy of Sciences 58277 , Changchun, People's Republic of China; 2 Huangpu Institute of Materials, Guangzhou, People's Republic of China; University of Maryland School of Medicine, Baltimore, Maryland, USA

**Keywords:** *Aureobasidium pullulans*, genome sequence, poly-malic acid

## Abstract

*Aureobasidium pullulans* Y2311-1 is a yeast-like fungus that can produce poly(β-L-malic acid). Here, we report the draft whole-genome sequence of the *A. pullulans* Y2311-1 strain, which includes 14 scaffolds (ranging in size from 0.02 to 3.52 Mb, 25.41 Mb total, 50.09% GC).

## ANNOUNCEMENT

As a biopolymer with excellent characteristics, including water solubility, biodegradability, and biocompatibility, poly(β-L-malic acid) (PMA) has attracted increasing attention for its broad prospective applications in food, pharmaceutical, biomedical, and environmental fields (1, 2). *Aureobasidium pullulans* Y2311-1, a commercially accessible strain, has been previously documented to exhibit remarkable productivity of PMA (3, 4). Here, we report the draft whole-genome sequence of the *A. pullulans* Y2311-1 strain. The analysis of the sequence may help us to gain further insight into the genetic information in *A. pullulans* Y2311-1 and to investigate strategies for further improving the strain.

*A. pullulans* Y2311-1 was purchased from Mingzhou Biotechnology Co., Ltd. (Ningbo, China) in October 2024. Cells from the glycerol stock stored at −80°C were inoculated and cultured in 50 mL of YM medium (0.5% peptone, 1% glucose, 0.3% yeast extract, 0.3% malt extract, and pH adjusted to 6.2) at 25°C for 20 h. The broth from multiple 50 mL flask cultures was then pelleted by centrifugation at 14,000 × *g* for 5 min, and genomic DNA was extracted using a cetyltrimethylammonium bromide-based protocol (5). High-molecular-weight genomic DNA (≥23 kb) was verified by visualization on an ethidium bromide-stained agarose gel.

For sequencing, a hybrid approach integrating Illumina and PacBio technologies was employed. Illumina library construction utilized the NEXTFLEX Rapid DNA-Seq Kit (NOVA-5144, Illumina), with fragmented DNA undergoing end repair, adenylation, adapter ligation, and PCR enrichment on the Illumina NovaSeq X Plus platform using the NovaSeq X Series 25B Reagent Kit (300 cycles, Cat. No. 20104706; Illumina). This generated 4,063,873 pairs of 150-bp raw reads, which were quality-controlled using fastp (version 0.20.0) via trimming low-quality bases, removing short reads, and discarding those with N bases. Specifically, bases with a quality score below 20 at the read tails were trimmed. A 10-bp sliding window was then employed; when the average quality score within the window dropped below 20, all subsequent bases starting from the window were removed. Reads that were shorter than 50 bp after undergoing these quality-trimming steps were discarded, ensuring that only high-quality sequences were retained for further analysis. Illumina reads were exclusively used for quality control of PacBio raw sequencing data and subsequent evaluation of genome assembly.

For PacBio sequencing, libraries were prepared using the SMRTbell prep kit 3.0 (102-182-700, PacBio). Genomic DNA was sheared to ~10 kb fragments with a Covaris g-TUBE, size-selected, and then ligated with hairpin adapters. For PacBio sequencing, libraries were prepared using the SMRTbell prep kit 3.0 (102-182-700, PacBio). Genomic DNA was sheared to ~10 kb fragments using a Covaris g-TUBE, followed by size selection performed with a Blue Pippin Instrument at a target range of ~10 kb prior to hairpin adapter ligation. Sequencing was performed on the PacBio Sequel IIe platform using the Revio Polymerase Kit (102-739-100, PacBio), yielding 92,390 raw reads with an N_50_ of 11,756 bp and an average sequencing depth of 42× coverage. Raw PacBio subreads were processed through SMRTLink 8.0’s ccs module (minFullPass = 3 and minPredictedAccuracy = 0.99) to generate high-fidelity (HiFi) circular consensus reads. The HiFi reads were sorted by barcode, saved in fastq format, and subsequently assembled using hifiasm v.0.19.5 with default parameters (6). The final assembly resulted in 14 scaffolds (3.52, 2.57, 2.5, 2.38, 2.19, 2.09, 2.08, 2.07, 1.91, 1.49, 1.34, 1.21, 0.04, and 0.02 Mb) with an N_50_ value of 2,193,234 bp. Genome annotation was performed using MAKER2 (version 2.32) with default parameters, identifying 9,707 protein-coding genes. The assembled genome exhibited a total size of 25,414,724 bp with a GC content of 50.09% and a genome coverage of 99.6% was achieved.

## Data Availability

This draft whole-genome sequencing and assembly project was deposited in GenBank (GCA_051942775.1). Sequence reads were deposited in the Sequence Read Archive (SRR34515462 and SRR36601877). The genome annotation files have been deposited to Zenodo (https://doi.org/10.5281/zenodo.17299326) (7).
